# A Latent Class Approach to Understanding Associations between Sports Participation, Substance Use, Dismissive Attitudes, and Sexual Violence Perpetration among High School Athletes

**DOI:** 10.1177/08862605211067005

**Published:** 2022-05-10

**Authors:** Katherine M. Ingram, Kathleen C. Basile, Ruth Leemis, Dorothy L. Espelage, Alberto Valido

**Affiliations:** 1 2331 University of North Carolina at Chapel Hill, Chapel Hill, NC, USA; 2 Division of Violence Prevention, National Center for Injury Prevention and Control, 1242 Centers for Disease Control and Prevention, Atlanta, GA, USA

**Keywords:** alcohol and drugs, cultural contexts, prevention, sexual assault, sexual harassment, youth violence, adolescence

## Abstract

Sexual violence (SV) among adolescents continues to be a major public health concern with numerous consequences. Research, predominantly with male collegiate samples, has suggested an association between sports participation and SV perpetration, and has included other important risk factors such as substance use and attitudes. However, more research is needed in this area among adolescents. The current study uses latent class analysis (LCA) to examine data- driven classes of high school student athletes (N = 665) engaged in three risk factor areas for SV: sport contact level, likelihood of substance use, and attitudes dismissive of SV. Once classes were enumerated and fit separately for male and female samples, pairwise comparisons were conducted on scores on two forms of SV (perpetration of sexual harassment and unwanted sexual contact) as a function of class membership. A 5-class solution was retained for both males and females. In the female sample, regarding SV—harassment, those most likely to perpetrate sexual harassment were those characterized by high likelihood of use of cigarettes, marijuana, alcohol, vape products, and those who played any type of sport. Too few females endorsed perpetration of unwanted sexual contact for pairwise comparisons to be conducted. For males, the classes most likely to perpetrate both forms of SV were those who were likely to endorse high likelihood to use of cigarettes, marijuana, alcohol, vape products, endorse attitudes dismissive of SV, and play any type of sport but especially high contact sports. These findings implicate high school athletic spaces as important venues for sexual violence prevention efforts.

## Introduction

Sexual violence (SV), defined as any form of unwanted sexual contact or sexualized attention without consent (e.g., lewd comments about one’s body, penetrative rape, unwanted sexual touch, and sexually explicit gestures; Basile et al., 2014), is a significant concern for the public’s health that often begins in childhood or adolescence. In the 2010 to 2011 school year, nearly half of all middle and high school students in the U.S. (56% of females and 40% of males) experienced a form of sexual harassment (Hill & Kearl, 2011). In 2019, about 10.8% (16.6% of females, 5.2% of males) of U.S. high school students reported unwanted sexual contact in the last year (Basile, Clayton et al., 2020). Data from the National Intimate Partner and Sexual Violence Survey (NISVS) reveal that among female victims of rape in youth, almost half (44%) reported an acquaintance as the perpetrator, and among them, 1 in 5 (21.6%) reported being raped by a friend. More than a third (35%) of the male victims in the same survey reported an acquaintance perpetrator (Merrick et al., 2018). These data suggest that peer-to-peer SV among adolescents merits empirical attention and evidence-based prevention efforts. Recently, SV has been recognized as a problem relevant to the sports industry (Raliance, 2017), with both empirical data (Lavigne, 2018) and several high-profile news reports (Phillips, 2015; Wagner, 2016). Given that 54% of high school students played at least one sport in 2017 (Kann et al., 2018), sports participation may be an ideal vehicle for prevention. These findings collectively highlight the need for research on the association between sports involvement and peer-to-peer SV in high school.

Athletic participation has long been associated with many positive outcomes for youth (see Eigenschenk et al., 2019; McKay et al., 2019 for recent reviews). However, a small but growing literature has also connected youth and young adult sports participation with SV perpetration (Basile et al., 2022; Gage, 2008; Humphrey & Kahn, 2000; Koss & Gaines, 1993; Locke & Mahalik, 2005). Most of this literature has focused on male perpetrators in university samples. In 2018, *Outside the Lines*, a sports news and entertainment program, conducted a study and found that collegiate athletes were about three times more likely than other students to be accused of sexual misconduct (Lavigne, 2018). Only a few studies have examined SV and sport in youth pre-college. McCauley and colleagues (2013) found that 5% of their sample of male high school athletes reported perpetrating physical/sexual abuse against dating partners between December 2009 and October 2010. One study of middle school students found that males who played high contact sports and females who played no or low contact sports were comparatively more likely to perpetrate SV later in high school (Basile, Clayton et al., 2020). In response to the dearth of literature on these associations in pre-college samples, the current study explores common profiles of SV perpetration risk factor endorsement among a sample of high school athletes, and associations between these profiles and SV perpetration. The current study contributes to the literature primarily in the following ways: (1) methodologically, as a person-centered analysis, (2) with a high school sample, and (3) more specific measures of sport involvement, substance use, and SV than previous studies.

## Theoretical Underpinnings of the Sexual Violence and Sport Association

Several scholars have conceptualized SV as an assertion of power (Uggen & Blacksone, 2004; Wood & Jewkes, 2001). This conceptualization is supported by common patterns of SV occurring across other power gradients, such as workplace management structures (Fitzgerald, 1993); Basile, D’Inverno et al., 2020), colonization (Smith, 2003), ability (Euser et al., 2016), race (West, 2014), and gender (Ellsberg et al., 2015). In this framework, having power privileges one’s desires and choices at the expense of a potential victim’s preferences (McGuinnes, 1993). These conditions may also make it difficult, unsafe, or impossible for a victim at the low end of a power gradient to say no or fight back, and facilitate powerful individuals engaging in SV without consequence (McGuinnes, 1993). These phenomena are illustrated in disparities in SV victimization. For example, Black and Indigenous women are often at highest risk of most forms of sexual and intimate partner victimization compared to women of other races (Smith et al., 2017). Further, Queer and Trans individuals are also more likely to be victimized than heterosexual and cisgender individuals (James et al., 2016; Walters et al., 2013). Further, each individual’s combination of identities and ideologies subjects them differentially to interlocking systems of power and oppression, such as white supremacy, patriarchy, homonegativity, and ablism (Crenshaw, 1989). So, everyone’s experience of privilege, power, and oppression as it relates to sexual violence may be different based on different combinations of identities, belief systems, environments, and experiences, but the overarching theme of SV as an assertion of power remains.

While SV occurs across and throughout peer networks, several key features of sports culture and climate give rise to favorable conditions for SV among these circles. Sports vary in their embedded aggression, but ultimately, they are a competition where one team must defeat another. In the most aggressive sports, this includes exerting physical power, such as bodily tackling (e.g., American football). Moreover, there is cultural socialization inherent to sports participation. Often, socialization includes exposure to traditional conceptions of masculinity. Traditional masculine socialization is a multi-faceted tool that upholds and reinforces patriarchy, the idea that men should hold power at the expense of women and other genders (hooks, 2010). It is also characterized by valuing toughness, aggression, antifemininity, and homonegativity (Kupers, 2005; Thompson & Pleck, 1986). This kind of socialization is highly present in the cultures and climates of sports in the U.S. Specifically, men’s athletic locker rooms are often spaces where conversations that demean women, foster homonegativity, and glorify violence occur and are celebrated, such that the colloquial phrase “locker room talk” has proliferated (Adams et al., 2010). One study of high school athletes found that male athletes, relative to females and male non-athletes, held significantly higher acceptance of rape myths (McMahon, 2015). Additionally, language used to describe winning against another team is often highly aggressive and sexist in nature, and often compares well-executed athletic moves to violent sexual acts (Adams et al., 2010). This practice glorifies SV by likening it to excelling in a game, a highly positive act. Additionally, one way that men may use SV to assert power as a function of being socialized this way is as a replacement for healthy emotional expression (Sugihara & Warner, 2002). Finally, the societies and communities in which sports are embedded also play a role in ascribing power to athletes. Despite often being manipulated or disempowered by institutions governing sports (e.g., NCAA, NFL; McLeran, 2017; Yearwood, 2018), socially, athletes often wield social power in their communities. Athletes are often made into celebrities and awarded copious attention and high wages. These assets provide relative social and economic power within one’s community (e.g., a campus; Hyman & Sierra, 2010; Ratten, 2015). Further, the gender gaps in pay, opportunity, and media coverage continue to provide evidence that cisgender male athletes are highly valued by society in the U.S (Cortes & Pan, 2016; National Collegiate Athletic Association, 2012).

## Additional Risk Factors

Several additional factors have been examined in the literature related to the association of sports involvement and SV perpetration, including the type and perceived importance of the sport. Based primarily on university student samples, the extant empirical literature on the sports-SV association has identified the importance of “centered-ness” and contact level. Messner (2002) proposed classifying sports as either centered (historically more valued by institutions) or marginalized (less valued by institutions). Examining categories of sports across the literature, the sports that involve the most aggressive contact are also the sports that are most often centered societally. One example of this convergence is that professional leagues that facilitate four of the most aggressive sports (based on collision contact) rank among the top five leagues for player salary (American Academy of Pediatrics, 2001; Statista, 2019). This phenomenon is also evident at other levels of sport play. For example, Gage (2008) conducted a study among NCAA athletes using Messner (2002)’s coding. This author found that males who played football (a high contact and centered sport) scored significantly higher on measures of hypermasculinity, negative attitudes toward women, and sexual aggression compared to males who were involved in marginal sports or no sports. McCauley and colleagues (2014) conducted a similar study in a sample of male high school athletes and found that those who played football, or a combination of football and basketball (high contact and centered sports), were more likely to report dating violence perpetration (including SV) than those who participated in other sports. Importantly, this study also controlled for attitudes regarding gender equality. These findings indicate that sports involvement and contact level may be uniquely responsible for variance in SV perpetration above and beyond what can be explained by traditional gender role attitudes.

Additionally, research on substance use, sports involvement, and SV during adolescence and young adulthood illustrates the complex relationships among these constructs. Kwan et al., (2014) found in a meta-analysis of 17 studies, that 14 studies showed a significant positive association between sports participation and alcohol use during adolescence. Koss and Gaines (1993) found that alcohol and nicotine use were the strongest predictors of SV perpetration, followed closely by sports participation, demonstrating the importance of substance use and the potential inter-relationship among substance use, sports involvement, and SV. A recent study found that for males, any individual or team sport participation predicted binge drinking, whereas females were only at-risk for binge drinking if they participated in team sports. Further, for both sexes, students who had higher social involvement (and for males, also high physical involvement) with their teams were more likely to binge drink (Boyes et al., 2017). These studies point to social dynamics related to socialization in cultures of youth sport communities, substance use, and SV acceptability. Though the literature suggests a positive association between sports involvement and alcohol use, and some indication that alcohol use by athletes may be related to SV perpetration, there is less agreement on the relationship among sports involvement, SV, and other substances. For example, Kwan and colleagues (2014) found significant negative associations among nicotine and marijuana use and sports involvement among adolescence. However, another study found that the negative association between team sport involvement and cannabis use was only true for females, not males (Boyes et al., 2017). The same study also found that sports involvement was associated with increased use of smokeless tobacco (e.g., chewing tobacco) for both males and females (Boyes et al., 2017). There is a dearth of literature on the relationship between marijuana use and SV; however, a recent meta-analysis suggests that marijuana use increases risk of dating violence (a construct highly related to SV) perpetration by 45% (Johnson et al., 2017). In recent years, “vaping” or e-cigarette use has risen among adolescents (National Institute on Drug Abuse, 2018). However, it has not yet been studied in relation to sport or SV.

Taken together, several studies point to associations among substance use, misogynistic attitudes, participating in physically aggressive sports, and SV perpetration, although these relationships vary by sample age, sex, and context. These relationships warrant further examination, especially among pre-college youth. It is critical to examine these associations through a developmental lens (throughout the literature) to identify points of transition into perpetration behavior, to inform prevention efforts. There is also very little literature addressing SV perpetration among female youth who play sports, and while they perpetrate SV significantly less often than males (Hill & Kearl, 2011; Ybarra & Thompson, 2018), the underpinnings of SV by female youth is also important to inform prevention.

## The Current Study

The present study examines associations among level of physical contact of sports participation, substance use, attitudes dismissive of SV, and SV perpetration of high school athletes by creating person-centered, latent classes of high school athletes’ involvement in each. We then examine associations between class membership and SV perpetration of male and female athletes involved in school sports. These analyses are expected to provide important information for prevention on how each person’s contact sports involvement operates within the context of other potential risk factors. We predict that, in each sample, the data will yield at least two latent classes. Additionally, we hypothesize that for both male and female athletes, the class(es) that report being more likely to use substances and more dismissive of SV will be more likely to have reported SV perpetration. Consistent with findings from Basile et al. (2022), among males, we also predict that classes with high involvement in high contact sports will be more likely to report SV perpetration, and among females, classes with high involvement in no or low contact sports will be more likely to report SV perpetration. Based on Basile et al. (2022), we anticipate low participation in high contact sports among females.

## Methods

### Participants

Participants (*N* = 665) were drawn from a larger sample of students participating in a randomized clinical trial evaluating *Sources of Strength* (Wyman et al., 2010). The clinical trial was advertised through school districts across a western U.S. state. To participate, schools had to agree to random assignment to treatment or waitlist control. All students in all classrooms in each high school were invited to participate. The current study used data from the 9 waitlist control schools at Wave 4 only, which was collected in the Spring of 2019, and includes only students enrolled in grades 10 through 12 (81% response rate). To be included in the current analyses, students had to have reported playing at least one sport at school in the current school year. Given that sports are generally split by sex assigned at birth and that participation occurs in these same-sex spheres, models for males (*N* = 348) and females (*N* = 317) were run separately. See Table 1 for sample demographics and values on study variables.

**Table 1.** Sample Characteristics of High School Athletes, Spring 2019, by Sex.

Variable	M (SD) or *n (%)*	Male	Female
	*N* = 665	*n* = 348	*n* = 317
*Sample demographics*			
Age	16.5 (.92)	16.5 (.96)	16.4 (.88)
Transgender	4 (0.6%)	3 (0.9%)	1 (0.3%)
Race/ethnicity			
Hispanic	371 (55.8%)	196 (56.3%)	175 (55.2%)
White	320 (48.1%)	153 (44.0%)	167 (52.7%)
American Indian/Alaskan Native	33 (5.0%)	17 (4.9%)	16 (5.0%)
African-American	28 (4.2%)	19 (5.5%)	9 (2.8%)
Asian	7 (1.1%)	4 (1.1%)	3 (0.9%)
Native Hawaiian/Pacific Islander	7 (1.1%)	3 (0.9%)	4 (1.3%)
Multiracial	110 (16.5%)	48 (13.8%)	62 (19.6%)
*Primary study variables*			
No-contact sports	266 (40.0%)	137 (39.4%)	129 (40.7%)
Some-contact sports	301 (45.3%)	113 (32.5%)	188 (59.3%)
High contact sports	432 (65.0%)	267 (76.7%)	165 (52.1%)
Cigarette use likelihood	50 (7.8%)	34 (10.1%)	16 (5.2%)
Alcohol use likelihood	240 (36.9%)	124 (36.6%)	116 (37.3%)
Marijuana use likelihood	209 (32.3%)	103 (30.4%)	106 (34.3%)
Vape use likelihood	279 (43.3%)	151 (44.9%)	128 (41.4%)
Attitudes dismissive of sexual violence	25 (3.9%)	23 (6.8%)	2 (0.6%)
Sexual violence– harassment**	126 (19.4%)	80 (23.8%)	46 (14.7%)
Sexual violence– contact**	20 (3.1%)	18 (5.4%)	2 (0.6%)

Note. Students were invited to select all of their racial identities if more than one was applicable. Therefore, the racial identity percentages do not sum to 100%. The “multiracial” category refers to the number of students who selected more than one racial identity, also counted above in the identity groups selected. Students were invited to select up to three sports in which they are currently participating, thus sport contact categories are not mutually exclusive and do not sum to 100%.

**Indicates count of individuals who disclosed one or more items on each perpetration scale.

### Procedure

The study was approved by four institutional review boards (IRBs). A waiver of parental consent was approved such that all parents received information letters and could opt their student out of participation by returning a form, calling the school, or emailing research staff. Eligible students were provided study information, and those who provided verbal assent were enrolled to complete online confidential surveys. Data collection occurred during regular class times with the supervision of two researchers in each classroom. Most students completed an online survey in English and translated paper surveys were used for Spanish-speaking students; the survey was also offered in braille in one school. All students were given resources after survey completion related to suicidal concerns, depression, and SV.

### Measures

#### Demographics

Each student was asked to report sex assigned at birth, whether or not they identified as transgender, age, and race/ethnicity. For race/ethnicity, students were asked to check all racial and ethnic identities that applied. Note that this manuscript is a secondary analysis of this dataset, and choices regarding demographic measurement were made based on needs and considerations of the primary analyses.

#### Sports Involvement

Students were asked “Did you play on a sports team that was affiliated with your school this school year?” Students could report up to three sports that they played from a drop-down list of 30 sports with an option for “no sports participation.” Each sport item was recoded into three separate variables that reflected the chosen sport’s contact category: no contact, low contact, or high contact. Contact categories are informed by policy statements made by the American Academy of Pediatrics Committee on Sports Medicine and Fitness (2001) and adapted to separate by levels of aggression (see Basile et al., 2022). For each contact category, students were assigned a “1” if they indicated participating in a sport in that category and a “0” if not.

#### Sexual Violence Perpetration

Sexual Violence perpetration was measured using a modified survey from the American Association of University Women Sexual Harassment Survey–Perpetration Scale (Hill & Kearl, 2011). This version consists of two subscales that emerged from factor analyses in a previous study of early adolescents: (1) SV—harassment and (2) SV—forced sexual contact (Espelage et al., 2012). Students were asked “How often, if at all, in the past SIX MONTHS have YOU done the following things to other students at school when they did not want you to?” The Sexual Harassment subscale consists of nine items which assess unwanted verbal commentary (e.g., “made sexual comments, jokes, gestures, or looks”), and the Forced Sexual Contact subscale consists of four items (e.g., “made them touch your private parts”; “forced them to have sex with you”). Response options are on a 5-point Likert-type scale ranging from 0 (*Never*) to 4 (*7 or more times*). Perpetration scales were binarized with “0” indicating never engaging in SV perpetration and “1” indicating any engagement during the past 6 months. Internal consistency estimates were high for Harassment Perpetration (α = .95) and Forced Sexual Contact Perpetration (α = .99). See Table 2 for rates of SV perpetration by demographic indicators.

**Table 2.** Percentage of Sexual Violence Perpetrators Within Each Demographic or Key Variable.

	Sexual Violence Harassment			Sexual Violence Contact		
	Male	Female	Total	Male	Female	Total
	*N* (%)	*N* (%)	*N* (%)	*N* (%)	*N* (%)	*N* (%)
*Sample Demographics*						
Age^a^ *r* (*p*)	0.07 (.188)	0.06 (.325)	0.06 (.119)	0.07 (.187)	0.03 (.660)	0.05 (.196)
Transgender	2 (66.7)	1 (100.0)	3 (75.0)	2 (66.7)	0 (0.0)	2 (50.0)
Hispanic	43 (21.9)	20 (11.4)	63 (17.0)	10 (5.1)	1 (0.6)	11 (3.0)
African-American	4 (21.1)	2 (22.2)	6 (21.4)	3(15.8)	0(0.0)	3(10.7)
American Indian/Alaskan Native	3 (17.6)	1 (6.3)	4 (12.1)	1 (5.9)	0 (0.0)	1 (3.0)
Asian	0 (0.0)	0 (0.0)	0 (0.0)	0 (0.0)	0 (0.0)	0 (0.0)
Native Hawaiian/Pacific Islander	0 (0.0)	0 (0.0)	0 (0.0)	0 (0.0)	0 (0.0)	0 (0.0)
White	35 (22.9)	33 (19.8)	68 (21.3)	5 (3.3)	2 (1.2)	7 (2.2)
Multiracial	8 (16.7)	9 (14.5)	17 (15.5)	1 (2.1)	1 (1.6)	2 (1.8)
No contact sports	28 (20.4)	25 (19.4)	53 (19.9)	7 (5.1)	2 (1.6)	9 (3.4)
Low contact sports	33 (29.2)	27 (14.4)	60 (19.9)	8 (7.1)	1 (0.5)	9 (3.0)
High contact sports	62 (23.2)	25 (15.2)	87 (20.1)	15 (5.6)	1 (0.6)	16 (3.7)
Likely to use cigarettes	10 (29.4)	3 (18.8)	13 (26.0)	5 (14.7)	1 (6.3)	6 (12.0)
Likely to use alcohol	41 (33.1)	29 (25.0)	70 (29.2)	10 (8.1)	2 (1.7)	12 (5.0)
Likely to use marijuana	33 (32.0)	27 (25.5)	60 (28.7)	8 (7.8)	2 (1.9)	10 (4.8)
Likely to vape	50 (33.1)	31 (24.2)	81 (29.0)	14 (9.3)	0 (0.0)	14 (5.0)
Attitudes dismissive of sexual violence	16 (69.6)	1 (50.0)	17 (68.0)	9 (39.1)	1 (50.0)	10 (40.0)

^a^indicates correlation between age and sexual violence perpetration.

#### Dismissiveness of Sexual Violence

The modified version of the National Institute of Justice Survey of Attitudes and Behaviors Related to Sexual Harassment (Taylor et al., 2008) assesses student’s dismissive attitudes toward SV. The six-item scale asks students to indicate how much they agree or disagree with each statement. Response options are on a 4-point Likert-type scale ranging from 0 (*Strongly Disagree*) to 3 (*Strongly Agree*). Example items include: “In my opinion... when boys make comments about girls’ bodies, girls should take it as a compliment” and “In my opinion... sexual harassment is just having fun.” This measure was binarized for current study. Students received a score of “1” to indicate higher dismissiveness of SV if their score was 2 standard deviations above the mean. Students below the mean were given a score of “0” to indicate lower dismissive attitudes. Internal consistency for the scale was high (α = .85).

#### Likelihood of Future Substance Use

Likelihood of future substance use was assessed with four questions asking students how likely it would be that they would engage in substance use behaviors during the next 6 months or next year. Students were asked “How likely are you in the next 6 months to …”: (1) “smoke cigarettes”; (2) “get drunk or very high on alcohol”; and (3) “use marijuana.” Response options were: 0 (*Not at all likely*); 1 (*Somewhat likely*); and 2 (*Very likely*). Additionally, students were asked “At any time in the next year, do you think you will use an electronic vapor product?” Response options were: 0 (*I definitely will not*), 1 (*I probably will not*), (2) (*I probably will*), 3 (*I definitely will*). For the current study, each question was binarized with “0” indicating no likelihood of future use and “1” indicating any likelihood of future use (defined as response options 2 through 3 for all questions). The validity of these measures was tested in a longitudinal sample of adolescents (*n* = 847) and it was found that intent to use was associated with actual drug use (Maddahian et al., 1988). Internal consistency for the scale was high (α = .98).

### Analytic Plan

To assess heterogeneity in profiles of sports participation by level of contact, substance use, and SV dismissiveness, we used a Latent Class Analysis (LCA) using the manual three step approach with the BCH auxiliary function in *Mplus* 8.3. We conducted separate analyses for male and female students. To determine the best fitting number of classes, we fit a series of six LCA models for each sample (six for males, six for females) where one class was added until fit was no longer acceptable. We then compared each model across several fit indices including −2 Log Likelihood (−2LL), Akaike Information Criteria (AIC), Bayesian Information Criteria (BIC), Sample-Size Adjusted BIC (SSBIC), Consistent Akaike Information Criteria (CAIC), Approximate Weight of Evidence Criterion (AWE), the Lo–Mendell–Rubin adjusted likelihood ratio test (LMRT), and the bootstrapped likelihood ratio test (BLRT; Masyn, 2013). Decreasing values among -2LL, AIC, BIC, SSBIC, CAIC, and AWE indicate improved model fit compared to the previous model (Nylund et al., 2007). The LMRT and BLRT examine whether adding an additional class is justified by testing the significance of the reduction in -2LL between a k class model and a k-1 class model (Lo et al., 2001). We also examined entropy, a measure of class separation ranging from 0 to 1 where, values above 0.80 are preferable (Grimm et al., 2016). Following class enumeration, control variables age and race/ethnicity were included as covariates and logits were used to fix classes prior to the outcome analysis (Nylund-Gibson et al., 2014).

To assess class associations with both SV scales, we used the auxiliary BCH approach (Asparouhov & Muthén, 2014). This method fixes the parameters of the latent classes to ensure that the measurement of classes is unaffected by the covariate (SV) values and uses a pseudo-class Wald’s chi-square test to assess all pairwise comparisons for mean differences on each SV scale between classes (Asparouhov & Muthén, 2014). We controlled for age and race/ethnicity in all models.

Missing data on all predictor and outcome variables was low, ranging from 2.39% to 3.15%. A chi-square test for missingness completely at random indicated that data were missing at random, *p* > .05.

## Results

### Latent Class Results for Female Athletes & Associations with Sexual Violence Measures

For females, fit indices yielded mixed evidence on the improved fit of the 5-class over the 4-class solution (Table 3). Negative 2LL and AIC values supported the 5-class solution, though BIC, SSBIC, CAIC, and AWE values supported a 4-class solution. Additionally, LRT was significant (*p* = .047), though BLRT was not (*p =* .162). Retaining the 5-class solution was justified by the adequate proportion of the sample in each class (all are greater than 9%), and the practical meaningfulness that the additional class yielded. Additionally, the increase in entropy from the 4-class to 5-class solution suggested improved separation between classes.

**Table 3.** Latent Class Analysis Model Fit Indices.

Females									
Model (K-class)	-2LL	AIC	BIC	SSBIC	CAIC	AWE	LMRT *p-value*	BLRT *p-value*	entropy
1-class	1949.28	1973.28	2018.39	1980.33	2030.19	2123.29	-	-	-
2-class	2383.64	2417.64	2481.54	2427.62	2498.54	2630.45	<.001	<.001	0.84
3-class	2342.09	2394.09	2491.82	2409.35	2517.82	2719.55	<.001	<.001	0.78
4-class	2284.90	2354.90	2486.46	2375.45	2521.46	2793.03	<.001	<.001	0.89
5-class	2264.73	2352.73	2518.12	2378.56	2562.12	2903.51	0.047	0.162	0.92
6-class	2238.92	2344.92	2544.14	2376.04	2597.14	3008.36	0.004	<.001	0.93
Males									
Model (K-class)	-2LL	AIC	BIC	SSBIC	CAIC	AWE	LMRT *p-value*	BLRT *p-value*	entropy
1-class	4197.15	4221.15	4267.38	4229.31	4279.39	4373.61			
2-class	2693.17	2727.17	2792.66	2738.73	2809.67	2943.16	<.001	<.001	0.83
3-class	2644.27	2696.27	2796.43	2713.95	2822.43	3026.59	<.001	<.001	0.90
4-class	2612.44	2682.44	2817.26	2706.23	2852.26	3127.09	<.001	<.001	0.92
5-class	2585.82	2673.82	2843.32	2703.74	2887.32	3232.81	0.037	0.050	0.93
6-class	2565.30	2671.30	2875.47	2707.34	2928.47	3344.63	0.004	0.090	0.94

**Figure 1.**
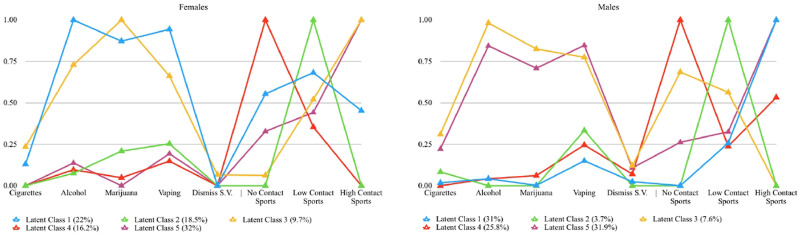
Average probabilities of each latent class indicator variable within each class.

Results for endorsement probability for each item by class can be found in Table 4 and Figure 1. About 22% of the female sample was in Class 1, scoring low on cigarette use propensity, but highest (of the classes) on likelihood to use alcohol and vape, and second most likely to use marijuana. This class was also low on SV dismissive attitudes, and moderately likely to have engaged in all types of sports. Class 2 was comprised of 18.5% of the sample, and was moderately likely to use marijuana and vape, low on SV dismissiveness, and high on low contact sports only. Class 3 included 9.7% of the sample. This class was highly likely to use alcohol and vape, and scored highest on likelihood of marijuana use compared to other classes. Though the chances of members being in the “high SV dismissiveness” category are overall low in this sample given the very low prevalence of dismissiveness among female athletes, Class 3 included the two individuals in the sample who did score at least 2 standard deviations above the mean on dismissiveness. Females in Class 3 were somewhat likely to play low contact sports and very likely to play high contact sports. Class 4, made up of 16.2% of the sample, was unlikely to use any substances, but somewhat likely to play low contact sports, and highly likely to play no contact sports. Finally, Class 5, the largest class (32.0% of the sample) scored low on all measures of substance use likelihood and SV dismissiveness. This class was somewhat likely to play no and low contact sports and highly likely to play high contact sports.

**Table 4.** Indicator Probabilities by Class.

	Females					Males				
	Class 1	Class 2	Class 3	Class 4	Class 5	Class 1	Class 2	Class 3	Class 4	Class 5
Cigarette use likelihood	0.130	0.000	0.235	0.000	0.000	0.017	0.083	0.310	0.000	0.222
Alcohol use likelihood	1.000	0.075	0.730	0.096	0.137	0.042	0.000	0.982	0.042	0.844
Marijuana use likelihood	0.872	0.209	1.000	0.048	0.000	0.002	0.000	0.825	0.061	0.709
Vape likelihood	0.944	0.253	0.662	0.148	0.193	0.150	0.333	0.775	0.246	0.847
Attitudes dismissive of sexual violence	0.000	0.000	0.066	0.000	0.000	0.024	0.000	0.121	0.070	0.107
No contact sports involvement	0.554	0.000	0.062	1.000	0.328	0.000	0.000	0.686	1.000	0.262
Low contact sports involvement	0.682	1.000	0.521	0.354	0.442	0.258	1.000	0.563	0.238	0.325
High contact sports involvement	0.453	0.000	1.000	0.000	1.000	1.000	0.000	0.000	0.533	1.000

Regarding SV–harassment, we found evidence of significant differences between classes in the overall analysis (*χ*^*2*^ = 11.55, *df* = 4, *p* = .021). Class 1 and Class 3 yielded the highest means on SV–harassment perpetration. Further, athletes in Class 1 were significantly more likely to report harassment perpetration than those in Classes 2, 4, and 5. See Table 5 for all comparisons. Too few female athletes endorsed perpetration of SV—contact for pairwise comparisons to be conducted.

**Table 5.** Class Comparisons of Sexual Violence Measures.

Females	Class 1	Class 2	Class 3	Class 4	Class 5	Sig. differences
SV – Harassment M (SE)	0.12 (.04)	0.01 (.01)	0.08 (.07)	0.02 (.01)	0.03 (.01)	Class 2, 4, 5 < class 1*
SV – Contact M (SE)	—	—	—	—	—	
Males	Class 1	Class 2	Class 3	Class 4	Class 5	Sig. differences
SV – Harassment M (SE)	0.07 (0.03)	0.13 (0.06)	0.55 (0.26)	0.11 (0.07)	0.33 (0.09)	Class 1 < Class 5**, Class 2 < Class 5^†^, Class 4 < Class 5*
SV – Contact M (SE)	0.05 (0.04)	0.08 (0.05)	0.41 (0.28)	−0.01 (0.01)	0.26 (0.09)	Class 1 < Class 5*, Class 4 < Class 5**

Note. Analyses control for race/ethnicity and age. Could not calculate class comparisons for SV – Contact among girls due to low incidence reporting. † *p* < .06, * *p* < .05, ** *p* < 0.01.

### Latent Class Results for Male Athletes & Associations with Sexual Violence Measures

For males, the 5-class solution fit best on measures of -2LL, AIC, SSBIC, LMRT, and BLRT (Table 3). Despite lower values on some measures for the 6-class solution, this solution included three very small classes, each including less than 5% of the sample. These classes were not practically meaningful nor justifiable. Entropy was sufficient in the 5-class model at 0.93.

Class 1 included 31% of the sample. These athletes in Class 1 were not likely to use any substances, reported low dismissiveness of SV, and were highly likely to play high contact sports. Class 2, which included 3.7% of the sample, was somewhat likely to vape, and very likely to play low contact sports. Class 3, which included 7.6% of the sample, was highly likely to use alcohol, marijuana, and vape. They scored the highest of all classes on dismissiveness of SV, and were somewhat likely to play no and low contact sports. Class 4 included 25.8% of the sample, and was characterized by a low likelihood of using substances, high likelihood of playing no contact sports, and a moderate likelihood of playing some or high contact sports. Finally, Class 5, which included 31.9% of the sample, was highly likely to use alcohol, marijuana, and vape, as well as play high contact sports. Class 5 also had the second highest score on SV dismissiveness. Probabilities of endorsement for each indicator can be found in Table 4 and Figure 1.

On both SV perpetration measures, overall chi-square tests of class differences were significant: SV-Harrassment (*χ*^2^ = 10.35, *df* = 4, *p* = .035), SV-Contact (*χ*^2^ = 15.92, *df* = 4, *p* = .003). Classes 3 and 5 scored highest on both SV perpetration types. Class 5 was significantly more likely to report harassment than Classes 1, 2, and 4 and significantly more likely to report forced contact than Classes 1 and 4. See Table 5 for all results of pairwise class comparisons.

## Discussion

This study used LCA to explore classes of contact sports participation, substance use, and dismissive attitudes of SV among a sample of high school athletes. Further, it examined differential prediction of two forms of SV perpetration as a function of class membership. Consistent with our hypothesis that data would yield at least two latent classes in each sample, we found that a 5-class solution fit best for both male and female athletes. Second, we expected that for both samples, the classes that scored highest on substance use and dismissiveness of SV would be more likely to report SV perpetration. We found that for females, two classes (Classes 1 and 3) characterized by high likelihood of using alcohol, marijuana, and vaping yielded the highest means on the SV-Harassment scale, and one of these classes (Class 1) was significantly more likely to report harassment perpetration than most other classes. Additionally, though very few female students endorsed attitudes dismissive of SV, Class 3 did include the highest scores on dismissiveness. Based on previous research (Basile et al., 2022), we also expected female classes with higher involvement in no/low contact sports would be more likely to report SV perpetration, but this hypothesis was not supported. Class 3 yielded the highest mean on the SV-Harassment scale for female athletes, characterized by high participation in high contact sports and only some participation in low contact sports. Among males, our hypotheses were supported across both SV measures: Classes 3 and 5 scored highest on both SV types and were characterized by higher likelihood of using marijuana, alcohol, and vaping, and having attitudes dismissive of SV. Also, males in Classes 3 and 5 were somewhat likely to play low contact sports, and those in Class 5 were highly likely to play high contact sports. Class 5 was significantly more likely to report harassment and contact perpetration than most other classes. These findings suggest that similar mechanisms are operating for both sexes with respect to high contact sports and sexual aggression, and that high poly-substance use in conjunction with high contact sport involvement may indicate increased risk for SV.

For both male and female athletes, poly-substance use, dismissive attitudes regarding SV, and high contact sports participation co-occurred in a large proportion of the sample, and membership in this class was associated with SV perpetration. While there were also classes of students who were equally likely to play sports but far less likely to use substances, these individuals were also far less engaged in SV. These findings suggest that sports involvement itself may not inherently be associated with sexual aggression, but rather only specific individuals or subcultures face increased risk, or it could be that the absence of substance use is important in explaining who does not perpetrate SV. These results illustrate heterogeneity among behavioral patterns of high school athletes. Like among non-athletes, substance use is highly connected to SV among athletes as well (Kwan et al., 2014). However, understanding risks specific to athletic environments allows for further specificity in understanding and targeting intervention. For example, how substances are introduced and used in the context of cross-grade sports team friendships are important target processes for coach- or school-disseminated intervention. The specific class findings by sex may inform how and for whom SV and substance use prevention efforts are focused depending on the type of sports of athletes. For example, female athletes in low contact sports may need substance use prevention efforts focused on vaping and alcohol use whereas female athletes in high contact sports might need marijuana use prevention. However, male high contact sport athletes endorsed plans to vape and use alcohol which suggests that coeducational prevention of SV and substance use might make the most sense with a combination of female athletes in low contact sports and male athletes in high contact sports, who are both endorsing vaping and alcohol use.

Notably, practice and performance environments for these high contact sports (e.g., basketball) are distinctly separated by sex, compared to sports like swimming or track where there may be more inter-player contact between males and females during practices and meets. One explanation may be that some of the students who use substances are self-selecting into contact sport environments, although this may not be the case for all athletes in high contact sports because some of them in this sample were classified as low substance users. Additionally, the group cohesion phenomenon may homogenize or polarize dominant cultures (e.g., rigid masculinity and aggressiveness) among teams, making it more likely or even adaptive for students who would not otherwise engage in substance use or SV perpetration to do so in the high contact sports context (Myers & Lamm, 1976; Rovio et al., 2009). Finally, though factor analysis suggested that the two SV constructs used are unique (rather than one combined construct), the two followed similar patterns in the current study, which is useful information for prevention efforts because it suggests that both contact and non-contact SV content can be included in the same intervention. This study’s findings suggest the need for longitudinal research to understand the development and confluence of contact sports participation, substance use and pro-violence attitudes over time and how they relate to SV perpetration.

This study contributes to our understanding of the association between contact sports participation and SV in numerous ways. First, it uses a high school sample, which is important given the large majority of previous scholarship is with college samples. Given the alarming rates of sexual assault on college campuses and other post-high school environments (Krebs, 2014), prevention efforts in high school are critical. Second, this study includes an examination of female and male athletes separately which is useful for tailoring prevention efforts. In addition, examining sexual harassment and forced contact acts separately is an important contribution, and can further help tailor prevention efforts. In this way, the current study expands on work done by Basile et al. (2022) by clarifying that female athletes most commonly report harassing behaviors instead of SV involving physical contact, whereas male athletes report both forms of SV (and both at higher rates, see Table 1). Further, for female athletes there is little variance in reported rates of SV harassment between classes, which may indicate that sexual harassment is either a somewhat normative component of sports teams or school culture. Finally, the current study examined key SV risk factors identified by previous literature (dismissive attitudes and substance use) in addition to contact sports involvement. It contributes to understanding of how contact sports involvement operates in the context of other potential risk factors. This analytic approach is centered around each person’s profile of indicators and how that predicts scores on SV outcomes, which better captures one’s realistic personal experience compared to a variable-centered approach. By not assuming independence between variables, we are better able to account for social processes that facilitate co-occurrence of phenomena such as polysubstance use and behavioral trends.

The critical importance of studying adolescence to understand the sports-SV link cannot be overstated. While most of the scholarship linking sports involvement to SV perpetration has been with college samples, SV begins earlier than college for many perpetrators (Espelage et al., 2012). Studies have shown that sexual harassment behaviors are prevalent in middle and high school (Basile et al., 2018; Espelage et al., 2012). Interrupting this pattern prior to adulthood (which may include collegiate or professional athletics) and preventing the potential for other forms of SV perpetration seems fruitful for youth development. Results of this study underscore the potential promise of the sports context for substance use and SV perpetration prevention with high school age youth and may also inform earlier prevention efforts in middle school. In fact, an emergent body of prevention research has used sports teams as a vehicle for sexual and other violence prevention (Miller et al., 2013). One evidence-based program, *Coaching Boys into Men (CBIM)*, engages athletic coaches to promote respectful and non-violent dating relationships among male athletes. Miller and colleagues (2013) found *CBIM* led to decreases in dating violence perpetration (including sexual). This seminal program provides evidence of the benefits of leveraging sports and mobilizing coaches as allies in prevention, but more evaluation research is needed that includes key risk and protective factors and examines these associations among female athletes.

The current results are constrained by several limitations. First, this sample is not nationally representative, so results may not be generalizable to students in other parts of the country or from demographic groups underrepresented in this sample. Additionally, this dataset did not adequately capture gender identity outside of binary sex assigned at birth and whether or not students had a transgender identity. These circumstances contribute to erasure of experiences of individuals who may be at highest risk of sexual violence and other forms of violence in sports, especially given the current legislation across the U.S. attempting to prohibit trans youth from participating in sports (Brassil, 2021). Given the aforementioned role of power in sexually violent encounters, an understanding of how gender and other socio-political identities (e.g., race, ethnicity, class, age, religion, ability, and sexual orientation) and the power dynamics that bring about and shape SV encounters among high school athletes is critical to explore. The current data did not allow for a deep analysis in this regard, and future work would be beneficial that centers non-binary youth voices and safety in examining how interpersonal violence manifests in athletics.

Second, the data presented are cross-sectional. While they offer important insights, they do not illuminate temporal relationships among the constructs (e.g., the socialization process of joining/staying on a team). Regarding measurement, all LCA indicators were binarized, prioritizing analytic functionality over data specificity. For measures of substance use likelihood, this method does not capture the amount of each substance students were likely to use nor frequency of likely use. Also, the measure of substance use asks about students’ self-reported likelihood of use rather than actual use. This method was mandated by a university IRB, as they considered drug use illegal behavior that would require breaking confidentiality to report identities of students who disclosed use to their schools. Also, while self-report of perpetration using behaviorally specific questions is considered best practice, it is likely an undercount of the true prevalence of SV given factors such as social desirability bias. It is also likely that in current social climates, SV dismissive attitudes are less acceptable than ever. Thus, reporting on attitudes dismissive of SV is also likely subject to influence of social desirability bias. Finally, LCA is useful for exploring common trends, but it still erases richness and specificity in individuals’ experiences. Though the 5-class solution fit both sexes best, there is still variance in likelihood of being in one’s assigned class.

Future research would be beneficial to better understand the athletic environments and socialization processes through which substance use, dismissive attitudes, and contact sports involvement co-occur, and why their co-occurrence is especially conducive to SV. Given the importance of substance use in our findings, additional research is warranted on how substances are introduced and used among athletes, and whether there are specific sport team characteristics that increase risk for substance use and later SV. Additionally, research is needed to expand the sports-SV perpetration model to go beyond the risk factors included in the current paper and develop a comprehensive model to explain the ways that the structure and common cultural components of organized sports in the U.S. gives rise to interpersonal violence, with explicit attention to non-binary youth. Given the multitude of benefits of youth sports outlined in the literature, future work will ideally examine the circumstances under which sports involvement can be leveraged to protect against SV perpetration and victimization, even in the context of risks. Given the powers that collectively govern organized sports (individual level (coaches, players), school/community levels (community culture and state and district policy), and federal level (federal legislation), efforts to protect against risk of violence in sports, especially for non-binary youth, would benefit from resources allocation to create systemic and sustained change.

This study’s findings add to the scholarship and inform current prevention efforts suggesting that high school and other adolescent athletic spaces can serve as opportunistic venues for SV prevention efforts, especially when prevention of substance use and pro-SV attitudes are addressed. The positive attributes of sports coupled with the high prevalence of sports team participation in high school and its cultural centrality within the U.S. suggest that sport can be leveraged for prevention of SV and other adolescent risk behaviors.
